# The problem of propranolol poisoning

**DOI:** 10.1002/bcp.70147

**Published:** 2025-07-03

**Authors:** Hayley Williams, Pardeep Jagpal, Euan Sandilands, Emma Morrison, Laurence Gray, Harry Krishna Ruben Thanacoody, Elisha Beg, Sally Bradberry, Robin Ferner

**Affiliations:** ^1^ National Poisons Information Service (Birmingham Unit) Birmingham UK; ^2^ National Poisons Information Service (Edinburgh Unit) Edinburgh UK; ^3^ National Poisons Information Service (Cardiff Unit) Cardiff UK; ^4^ National Poisons Information Service (Newcastle Unit) Newcastle UK; ^5^ Church Street Medical Practice Oxfordshire Clinical Commissioning Group Oxford UK; ^6^ West Midlands Poisons Unit Sandwell and West Birmingham NHS Trust Birmingham UK; ^7^ University of Birmingham Birmingham UK

**Keywords:** anxiety, overdose, prescribing, primary care, propranolol

## Abstract

**Aims:**

Propranolol is licensed in the UK and elsewhere to relieve symptoms of anxiety. In overdose, propranolol poisoning can be serious, difficult to treat and potentially fatal. This paper describes cases of intentional propranolol overdose reported to the UK National Poisons Information Service in order to raise awareness of the risk of harm following propranolol overdose and support safer prescribing.

**Methods:**

This study reviews enquiries to UK Poison Centres involving intentional overdoses of propranolol requiring hospital attendance and reported between 1 January 2022 and 31 December 2023.

**Results:**

There were 444 enquiries about 363 intentional propranolol overdoses in 359 different patients (248 [69%] women). Median age [interquartile range, IQR] was 32 [22–45] years (*n* = 355); 52 (14%) patients were <18 years old. The indication was known in 121 (33%) cases, and was anxiety in 111. In 43/61 cases where propranolol and an antidepressant were co‐prescribed, patients took both in overdose. The outcome was confirmed in 159 (44%) exposures; 110 patients recovered completely, 13 had ongoing features, one had permanent sequelae, and 35 died.

**Conclusions:**

Overdose patients were often prescribed propranolol for anxiety, and many developed systemic toxicity. Despite intensive treatment, many patients still die from poisoning with propranolol alone or in combination with other agents. Given the dangers of propranolol in overdose, prescribers based in primary care settings should recognize that using propranolol to manage anxiety can be dangerous.

**What is already known about this subject**

- Propranolol can attenuate the physical symptoms associated with performance anxiety, but has not shown efficacy in anxiety states.
- Propranolol is widely prescribed in UK general practice, and prescribing rates are increasing.
- We wished to see whether propranolol poisoning caused a significant clinical problem.

**What this study adds**

- Severe propranolol poisoning most often affects women and those who have been prescribed propranolol for anxiety.
- Propranolol is often taken in overdose with a co‐prescribed antidepressant.
- It may be best not to prescribe propranolol for anxiety if there is a prior history of overdose.

## 1 INTRODUCTION

Propranolol is a non‐selective β‐adrenoceptor (β1‐ and β2‐adrenoceptor) antagonist, and therefore reduces heart rate and tremor in thyrotoxicosis and anxiety states.^1^, ^2^ These effects have led to a ban on taking propranolol and related drugs in sports such as archery and darts.^3^ β‐adrenoceptor antagonists can attenuate the effects of performance anxiety (‘stage fright’) in musicians,^4^, ^5^ but not necessarily subjective feelings of anxiety.^6^ The indication was extrapolated to generalized anxiety disorders without thorough investigation. A systematic review of the use of propranolol to treat anxiety disorders 50 years after initial studies concluded that ‘the quality of evidence for the efficacy of propranolol at present is insufficient to support the routine use of propranolol in the treatment of any of the anxiety disorders’,^7^ and a more recent review concluded that, while β‐adrenoceptor antagonists may reduce symptoms of performance anxiety, they are not recommended for other anxiety disorders.^8^ In the UK, propranolol's licensed indications still include ‘relief of situational anxiety and generalized anxiety symptoms, particularly those of somatic type’.^9^ However, the UK National Institute for Health and Care Excellence guidelines for the management of generalized anxiety disorder do not recommend propranolol or other β‐adrenoceptor antagonists.^10^ Oral propranolol tablets of 10, 40, 80, and 160 mg and modified‐release propranolol capsules of 80 and 160 mg are available in the United Kingdom.

The uncertain efficacy of propranolol in anxiety contrasts with the well‐characterized dangers of propranolol poisoning. Propranolol is lipophilic, crosses the blood–brain barrier and causes both β‐adrenoceptor antagonism and voltage‐gated sodium channel (Na_V_ ) blockade. These effects cause hypotension, bradycardia, heart block and severe cardiac depression, leading to ventricular arrhythmias, cardiac arrest and death. Coma and seizures can occur.^11^

The UK National Poisons Information Service (NPIS) provides information and evidence‐based management advice to NHS healthcare professionals managing cases of poisoning. This is delivered through an online database, TOXBASE®, and a 24‐h telephone advice service. NPIS has tracked overdoses involving propranolol since 2019^12^, ^13^, ^14^, ^15^; a fifth resulted in moderate, severe or fatal poisoning (*n* = 93/410, 22%).^12^, ^13^, ^16^ In a 5‐year review of 1925 propranolol exposures reported to the NPIS, at least 46 overdoses (2.4%) resulted in death.^14^, ^15^

Telephone enquiries to the UK National Poisons Information Service (NPIS) were analysed to identify the potential burden of propranolol poisoning.

## 2 METHODS

### 2.1 Design

The study was an observational case series of patients based on enquiries to the National Poisons Information Service. Where informative in the context of the clinical service, callers were asked specific questions (Table 1).

**TABLE 1:** Pre‐specified questions.

Has the patient got a history of taking overdoses?
On this occasion, how many tablets were ingested and when?
Is the propranolol the patient's own medication?
If so, what is the indication for its use?
What is the patient's usual dose?
What strength tablets?
Are they sustained release?
How many tablets are prescribed/dispensed at a time (if known)?
Other drugs prescribed therapeutically?
Other drugs taken in overdose (and doses if known)?

### 2.2 Setting

All telephone enquiries to the NPIS, which provides expert advice to NHS healthcare staff in the United Kingdom, are recorded in a single structured electronic database, UKPID (UK Poisons Information Database).

The data collected include date and time of enquiry, patient's age, sex and substances reported to be taken including co‐ingestants, route of exposure, clinical features and recommended management. Information scientists answering NPIS telephone enquiries also collect additional information deemed clinically relevant to patient care. A healthcare professional may contact the service at any time a patient is, or may be, poisoned, so the enquiry logs can only ever provide a partial account of the poisoning episode, and the data recorded may be incomplete.^17^

The clinical features are graded using the Poisoning Severity Score (PSS),^16^ which runs from None = 0 (‘No symptoms or signs related to poisoning’), through Mild = 1 (‘Mild, transient and spontaneously resolving symptoms’), Moderate = 2 (‘Pronounced or prolonged symptoms’), and Severe = 3 (‘Severe or life‐threatening symptoms’) to Fatal = 4 (‘Death’).

### 2.3 Analysis

We surveyed hospital enquiries involving intentional overdoses of propranolol reported to the NPIS between 1 January 2022 and 31 December 2023. Data were extracted from UKPID and retrospectively analysed in Microsoft Excel® (data included patient age, sex and PSS) and, where available, answers to specific queries (Table 1). Results are expressed as median [interquartile range]. The confidence interval of a proportion was calculated using Vassarstats.net.^18^ In addition, we extracted the total number of enquiries received by the NPIS involving propranolol since 2017.

### 2.4 Nomenclature of targets and ligands

Key protein targets and ligands in this article are hyperlinked to corresponding entries http://www.guidetopharmacology.org, and are permanently archived in the Concise Guide to PHARMACOLOGY 2023/24.^19^, ^20^

## 3 RESULTS

**FIGURE 1:**
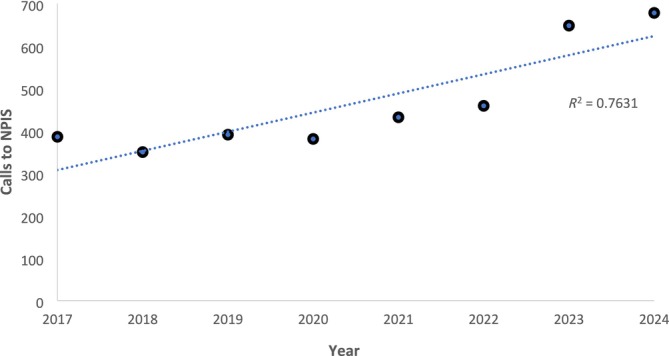
Calls involving propranolol to the NPIS by year.

Between 2017 and 2024, the number of enquiries (from all sources) to the service about propranolol increased linearly from 386 to 678 (*r* = 0.874, *P* = 0.005) (Figure 1). During the study period (2022–2023) there were 444 hospital enquiries involving 359 different patients (female = 248, male = 110, sex unknown = 1) aged 12–87 years who took 363 intentional propranolol overdoses. There were 149 overdoses in 2022 and 214 in 2023. In 30 cases, it was recorded that slow‐release propranolol capsules had been taken. Baseline characteristics and outcome of the cases are shown in Table 2. The median recorded dose associated with different grades of toxicity is shown in Table 3 for both all overdoses and overdoses where the only drug taken was propranolol.

**TABLE 2:** Baseline characteristics and outcome in 359 patients who took 363 intentional overdoses that included propranolol. Results are given as median [interquartile range] and range, or as percentages.

**Age (*n* = 355)**	32 [22–45] (range 12–87) years
**Sex**	
Female	248 (69%)
Male	110 (31%)
Unknown	1 (0.3%)
**All propranolol overdoses (*n* = 363)**	
Propranolol + other drugs	289 (80%)
Propranolol alone or with alcohol	74 (20%)
**Dose (*n* = 247)**	840 [360–2240] (range 30–26 880) mg
**Propranolol prescription**	
Known prescription to patient	200 (55%)
Indication known (*n* = 121)	
Anxiety	111 (92%)
Migraine	7 (6%)
Hypertension	3 (2%)
Not prescribed to the patient	38 (10%)
Unascertained	125 (34%)
**Confirmed outcome (*n* = 159)**	
Complete recovery	110 (69%)
Ongoing features	13 (8%)
Permanent sequelae	1 (0.6%)
Death	35 (22%)

**TABLE 3:** Median (IQR) dose of propranolol ingested compared against Poisoning Severity Score.

Poisoning Severity Score	Cases, *n* ^a^	Median dose [IQR] (mg)	Propranolol alone or with alcohol, *n* ^a^	Median dose [IQR] (mg)
**None**	28 (22)	600 [280–800]	8 (8)	800 [620–1425]
**Minor**	71 (58)	560 [240–1120]	15 (15)	1000 [530–2240]
**Moderate**	86 (63)	640 [290–1600]	15 (13)	1600 [600–3200]
**Severe**	138 (83)	1800 [560–3920]	23 (17)	3200 [2800–4080]
**Fatal**	35 (18)	2120 [1220–3540]	12 (4)	2800 [1900–5700]

^a^ The figures represent total number of cases (number of cases in which the dose was known).

Of the 74 (20%) patients who took propranolol alone (or with alcohol), 50 patients (68%) developed moderate (*n* = 15), severe (*n* = 23) or fatal (*n* = 12) toxicity. The ingested dose, where known, ranged from 30 to 12 000 mg. The median recorded dose associated with moderate toxicity was 1600 [600–3200] mg (*n* = 13), severe toxicity was 3200 [2800–4080] mg (*n* = 17) and fatal toxicity was 2800 [1900–5700] mg (*n* = 4).

In at least 38 (10%) overdoses, propranolol had not been prescribed to the patient; 17 of these patients were <16 years old; 11 were aged 16–25 years and 10 were aged 26–73 years. The median age was 16 [14–26] years. In 125 (34%) overdoses it was not ascertained whether propranolol had been prescribed to the patient or someone else.

Information about other drugs prescribed to the patient was available in 180 (50%) cases; in 128/180 (71%) cases an antidepressant was also prescribed. In 103/128 (80%) cases, the antidepressant was taken as part of the overdose, most commonly sertraline, venlafaxine or fluoxetine.

The total dose of propranolol dispensed was known in 26 (7%) cases. A toxic dose (4.5 mg/kg according to TOXBASE®, taken to be 315 mg total) had been dispensed to the patient in 24 (92%) cases; a potentially fatal dose (2 g according to TOXBASE®) had been dispensed to 10/24 (42%) patients. At least 10/24 patients had a history of overdose.

### 3.1 Sex differences

Totals of 110 males (111 exposures) and 248 females (251 exposures) took intentional overdoses of propranolol. A fatal outcome was more common in women (29/251 *vs*. 5/111; *P* = 0.049, Fisher's exact test). Characteristics and outcomes are compared between the sexes in Table 4.

**TABLE 4:** Differences between males and females who took propranolol overdoses.

	Males	Females
**Number of patients**	110	248
**Number of exposures**	111	251
**Age (years)**	33 [23–46] (range 12–80)	32 [22–44] (range 12–87)
**Dose (mg)**	800 [440–2240] (*n* = 75)	960 [315–2260] (*n* = 172)
**Propranolol prescription**		
**Known prescription to patient**	50	150
Anxiety	31	80
**Previous overdose recorded**	27 (24%)	74 (29%)
**Poisoning severity**		
Moderate	26 (23%)	60 (24%)
Severe	47 (42%)	91 (36%)
Fatal	5 (5%)	29 (12%)

### 3.2 Indications

Propranolol was prescribed for anxiety in 111 patients (80 female and 31 male) and the dose ingested, known in 85 cases, was 960 [480–2000] mg. For 28 patients this was their first overdose, but at least 47 (42%) had taken an intentional overdose previously. An antidepressant was prescribed in addition to propranolol in 61/111 cases, and 43 took both propranolol and an antidepressant in overdose.

### 3.3 Fatal cases

A fatal outcome was recorded in 35 patients (fatality rate 9.6%, confidence interval [CI] 6.9%–13%) with a median age of 34 [27–45] (range 20–58) years. Fifteen deaths were recorded in 2022 and 20 in 2023. The reported ingested dose, available in 18 cases, was 2120 [1220–3540] mg. Females accounted for 29/35 fatalities (83%); 13 had a previous history of overdose and seven were known to be prescribed propranolol for anxiety (*n* = 6) or migraine (*n* = 1).

### 3.4 Patients less than 18 years old

A total of 52 intentional propranolol overdoses were recorded in patients aged <18 years (35 female and 17 male). The propranolol prescription was for the patient in 17 cases, for another person, usually the patient's mother, in 23 cases, and not ascertained in 12 cases.

The dose ingested, known in 42/52 cases, was 560 [305–1120] mg. Although no patient <18 years died, a poisoning severity score of ‘moderate’ was recorded in 12 cases, and ‘severe’ in 14 cases, accounting for 50% of intentional propranolol overdoses in this age group.

## 4 DISCUSSION

### 4.1 Summary

NPIS is commonly, and increasingly, called about intentional propranolol overdose. Most patients are women, and over half take propranolol prescribed to the patient, most commonly for anxiety. The severity of poisoning is dose‐related, and most intentional propranolol overdoses in our cohort resulted in moderate toxicity or worse, with a case fatality rate of 9.6%.

Females were more likely to take an intentional overdose of propranolol, and if they did, were more likely to have been prescribed propranolol for anxiety, take larger quantities in overdose, and die as a result of overdose. Where known, almost all prescriptions were for quantities of propranolol that were potentially toxic, and in almost half, potentially fatal.

### 4.2 Strengths and limitations

Our study shares the limitations of most poison centre studies.^21^, ^22^, ^23^ The number of enquiries made to the NPIS about a medicine does not correspond with the number of patients exposed, many of whom are managed without the need for expert telephone advice, since clinicians can and often do manage patients without assistance, or by reference to the web‐based TOXBASE® information system maintained by the NPIS. Some patients may be the subject of more than one enquiry. The telephone enquiries are therefore biased towards more serious cases. There is no direct contact between the patient and the NPIS, which has to rely on details obtained and passed on by the enquirer, often in the context of medical urgency. There are therefore inevitably missing data that can distort the results, and some data, for example on past medical history or concomitant illness, may not be captured. Analytical confirmation of exposure and exclusion of other potential toxins is not available as this is not performed as part of the routine care of patients after propranolol overdose. Despite attempts to follow up all the patients, an outcome was confirmed in less than half of cases discussed. We were not able to access Coroners' Inquest reports on fatal cases.

### 4.3 Comparison with existing literature

Our study is consistent with other data suggesting that propranolol overdose is an important cause of serious and fatal poisoning in the United Kingdom, and the incidence may be increasing. Over the years 2017–2024, the number of calls to the service about propranolol increased and the number of patients known to have died from overdoses involving propranolol rose from 7 in 2017 to 20 in 2023, with a total of 83 deaths.^13^, ^14^, ^15^, ^16^, ^24^

The Office of National Statistics analysed drug‐poisoning deaths where propranolol was mentioned on the death certificate annually from 1993, when there were 16 deaths, to 2017, when there were 52 deaths, to 2022, when there were 76 deaths.^25^, ^26^

The Healthcare Safety Investigation Branch (now the Health Services Safety Investigation Body [HSSIB]) reported in 2020 the potentially under‐recognized risk of fatal harm from propranolol.^27^ Coronial toxicology data also indicate an important increase in the detection of propranolol in suicidal poisoning.^28^ Of 4176 suicides with postmortem toxicology, anxiety featured in 8.6%; in the 297 involving propranolol, anxiety featured in 22% (*P* < 0.05). The proportion of suicides in which propranolol was detected rose from 3.4% in 2010 to 12.3% in 2021. Several Coroners' Letters to Prevent Future Deaths have referred to propranolol‐related deaths.^29^

In Denmark, it has been suggested that it is ‘difficult to argue that prescribing propranolol in performance anxiety is rational’.^30^ A Danish register study found significant correlation between students being prescribed propranolol for performance anxiety and increased incidence of attempted suicide.^31^

A further concern in Denmark was the dispensing of 10 mg propranolol tablets (the common dosage for performance anxiety) only in pack sizes of 250 tablets (2500 mg), a toxic and potentially fatal quantity.^12^ This was underlined by several cases of younger people (aged 14–30) ingesting between 120 and 249 tablets.^30^ The lowest recorded fatal dose of propranolol alone in our series was just 1600 mg in a 40‐year‐old woman, who was prescribed propranolol 40 mg three times a day for anxiety.

### 4.4 Implications for research and practice

Despite these increasing safety concerns, propranolol prescribing in UK general practice continues to increase, from 389 million tablets in 2021 rising steadily to 480 million tablets in 2024.^32^ The rise in the use of propranolol may in part be due to the increasing, and understandable, reluctance to prescribe benzodiazepines. It has been recommended since 1988 that benzodiazepine treatment be limited to 2–4 weeks' duration,^33^ so that seems not to be the entire explanation.

A significant reason for the continued prescribing of medicines for anxiety in UK general practice is the disjunction between the NICE recommendations, which emphasize psychological interventions for the initial management of generalized anxiety disorders; and the lack of access to NHS psychology.^34^ In a recent UK study, which sought to understand when and why GPs prescribe beta‐blockers for people with anxiety, safety‐driven prescribing was identified as a major factor.^34^ Many GPs viewed beta‐blockers as ‘low‐risk,’ particularly in young adults. Some GPs viewed beta‐blockers as an alternative to benzodiazepines, acting quickly and not leading to dependence. Many GPs were content for a ‘patient‐led’ approach, with patients managing their own dose and frequency, with less need for GP input. When asked about risks or contraindications for prescribing beta‐blockers, most GPs talked about the risks for patients with anxiety who also had asthma, low blood pressure, were older or were pregnant. None of the GPs surveyed mentioned any concerns relating to overdose.

## 5 CONCLUSION

UK poison centres are frequently contacted for advice on the management of propranolol poisoning. In our cohort, overdose patients were often prescribed propranolol for anxiety, and many developed systemic toxicity. Despite intensive treatment, poisoned patients still die from poisoning with propranolol alone or in combination with other agents. There is no clear evidence of benefit from propranolol treatment in anxiety, except perhaps performance anxiety. Given the dangers of propranolol in overdose, careful assessment of the appropriateness of its prescribing is necessary. In some cases, particularly in patients with a history of previous overdoses, there are good reasons for avoiding its use entirely.

## AUTHOR CONTRIBUTIONS

HW designed the study. HW, PJ and RF undertook data analysis and interpretation, and drafted the initial manuscript. EB and SB reviewed and amended the manuscript. LG, HKRT, ES and SB gave permission for data acquisition as NPIS data controllers and critically reviewed the manuscript. All authors reviewed and approved the final manuscript for submission.

## CONFLICT OF INTEREST STATEMENT

The authors have no conflicts of interest to declare.

## DISCLAIMER

A subset of these findings has been presented as a conference abstract at the annual congress of the European Association of Poison Centres and Clinical Toxicologists, Munich, Germany, May 2024.

## Data Availability

Data sharing is not applicable to this article as no new data were created or analyzed in this study.
